# CsCOI1 regulates plant growth and defense in citrus

**DOI:** 10.1093/hr/uhaf174

**Published:** 2025-07-07

**Authors:** Gang Hu, Bing Liu, Kun Yang, Wei-Kang Zheng, Yi Zhang, Cai-Xia Teng, Duo-Yi Huang, Ruo-Hao Yan, Michitaka Notaguchi, Munenori Kitagawa, Zong-Cheng Lin, Qiang Xu

**Affiliations:** National Key Laboratory for Germplasm Innovation & Utilization of Horticultural Crops, Huazhong Agricultural University, Shizishan street No.1, Wuhan 430070, China; National Key Laboratory for Germplasm Innovation & Utilization of Horticultural Crops, Huazhong Agricultural University, Shizishan street No.1, Wuhan 430070, China; National Key Laboratory for Germplasm Innovation & Utilization of Horticultural Crops, Huazhong Agricultural University, Shizishan street No.1, Wuhan 430070, China; National Key Laboratory for Germplasm Innovation & Utilization of Horticultural Crops, Huazhong Agricultural University, Shizishan street No.1, Wuhan 430070, China; National Key Laboratory for Germplasm Innovation & Utilization of Horticultural Crops, Huazhong Agricultural University, Shizishan street No.1, Wuhan 430070, China; National Key Laboratory for Germplasm Innovation & Utilization of Horticultural Crops, Huazhong Agricultural University, Shizishan street No.1, Wuhan 430070, China; National Key Laboratory for Germplasm Innovation & Utilization of Horticultural Crops, Huazhong Agricultural University, Shizishan street No.1, Wuhan 430070, China; National Key Laboratory for Germplasm Innovation & Utilization of Horticultural Crops, Huazhong Agricultural University, Shizishan street No.1, Wuhan 430070, China; Bioscience and Biotechnology Center, Nagoya University, Furo-cho, Chikusa, Nagoya 464-8601, Japan; Department of Science, Kyoto University, Kitashirakawa Oiwake-cho, Sakyo, Kyoto 606-8502, Japan; National Key Laboratory for Germplasm Innovation & Utilization of Horticultural Crops, Huazhong Agricultural University, Shizishan street No.1, Wuhan 430070, China; National Key Laboratory for Germplasm Innovation & Utilization of Horticultural Crops, Huazhong Agricultural University, Shizishan street No.1, Wuhan 430070, China; National Key Laboratory for Germplasm Innovation & Utilization of Horticultural Crops, Huazhong Agricultural University, Shizishan street No.1, Wuhan 430070, China; Hubei Hongshan Laboratory, Shizishan street No.1, Wuhan 430070, China

## Abstract

Jasmonates (JAs) play essential roles in plant development and defense. JA perception and responses remain elusive in citrus. Here, we identified core components for JA perception in citrus and elucidated transcriptional changes associated with JA signaling in growth and defense. We showed the F-box protein CORONATINE INSENSITIVE1 (COI1) in citrus is a JA receptor, as *Cscoi1* mutants are insensitive to methyl jasmonate and CsCOI1 interacts with CsJAZs in the presence of JA-Ile. CsCOI1-mediated JA signaling represses shoot growth while enhancing resistance to insects. Consistently, CsCOI1 represses the expression of growth promoting genes such as *PIF7*, while upregulating genes related to defense metabolites in terpene and flavonoid pathways. Additionally, JA signaling antagonizes salicylic acid (SA) signaling at the transcriptional level and promotes susceptibility to citrus canker disease. This study highlights the role of JA signaling in balancing growth and resistance to biotic stress in citrus, revealing critical trade-offs for consideration in precision citrus breeding.

## Introduction

Plants must grow for reproduction while also defending themselves for survival in nature. However, an inverse relationship between growth and defense is often observed, with higher defense generally resulting in a growth penalty [1, 2]. Although resource limitation is a common explanation for this growth-defense trade-off, another factor from the plants side is their temporal adaptation to the changing environment [3]. Recent studies on the molecular mechanisms underlying the plant growth-defense trade-off suggest that plants tend to limit their growth to restrict proliferation of their enemies. These findings highlight the prominent role of antagonistic crosstalk between plant hormones in regulating the allocation of growth and defense [3–5].

Jasmonates (JAs) are lipid-derived plant hormones that promote plant defense against insects and necrotrophic pathogens while restricting plant growth [1, 6, 7]. In *Arabidopsis*, JAs are known to be perceived by CORONATINE INSENSITIVE1 (COI1), the F-box subunit of an E3-ubiquitin ligase that polyubiquitinates JASMONATE ZIM DOMAIN (JAZ) proteins [8]. In the absence of JAs, JAZ transcriptional repressors inhibit the activity of MYC transcription factors (TFs) via direct protein–protein interactions [9, 10]. JA signals accumulate in response to a fluctuating environment and act as chemical ligands promoting the interaction of COI1 and JAZs, thus leading to degradation of JAZs and release of MYCs [11–13]. Since MYCs target hundreds of TFs, large transcriptional reprogramming is activated during JA responses [14, 15]. These transcriptional changes allocate resources to high defense and slow down growth [16, 17]. Although this JA signaling pathway has been studied in several herbaceous plants [18–21], the JA perception mechanism in woody plants remains under-investigated.

In *Arabidopsis* and rice (*Oryza sativa*), studies have shown that elicitation of JA responses results in lower accumulation of the plant growth-promoting hormone gibberellins (GAs) and inhibition of the GA response [4, 22]. In the GA signaling pathway, DELLA proteins are key repressors that are degraded in response to the GA signal. Under basal conditions, the activity of growth-related regulators, such as PHYTOCHROME-INTERACTING FACTORS (PIFs), is suppressed by DELLA repressors [23]. JAZs interact with DELLA proteins and interfere with their function [22, 24]. Under JA-related defense conditions, JAZs are degraded by COI1, and thus more DELLAs are available for growth suppression [22, 24]. Plants also repress the JA signaling pathway when growth is the primary goal. For instance, a low ratio of red to far-red light can induce shade avoidance response. Under this condition, stem elongation is necessary for light competition. Therefore, DELLAs are depleted to allow fast growth, while JAZs accumulate to limit the JA response [25]. Additionally, more active PIFs accumulate and induce the reduction of bioactive JA [26]. In most other plants, the mechanism for the JA-regulated trade-off between plant growth and defense remains elusive.

Citrus trees are important fruit crops growing across the tropical and subtropical regions of the world [27]. The tree architecture and resistance greatly influence the quality, yield, and cost of the citrus industry. In major crops, uncoupling the growth and defense trade-off is often desired for breeding superior crops with high yield and no defense penalty [3, 28]. In fruit trees, the JA-mediated growth-defense trade-off appears to be directly utilized without modification, as both dwarf tree architecture and high resistance are important traits in fruit tree breeding [29, 30]. Nonetheless, its further application is hindered due to the limited understanding of core mechanisms of JA signaling and responses in woody plants.

Here, we investigated the functions of JAs in regulating growth-defense trade-off in citrus. Through genetic analysis and biochemical assays, we characterized the core components for JA perception and response in citrus. By analyzing CsCOI1 genome editing mutants, we found that JA signaling promotes resistance to insects while repressing the growth of citrus, supporting the idea of obtaining dwarf trees with high resistance to insect herbivores through the upregulation of JA response. Furthermore, we showed that CsCOI1 promotes susceptibility to citrus canker disease while repressing the transcription of genes in the salicylic acid (SA) signaling pathway. This study highlights the role of JA signaling in balancing growth and resistance to biotic stresses in citrus and provides insights into breeding strategies through the fine-tuning of JA-related genes.

## Results

### JA signaling negatively regulates plant growth of citrus

**Figure 1:**
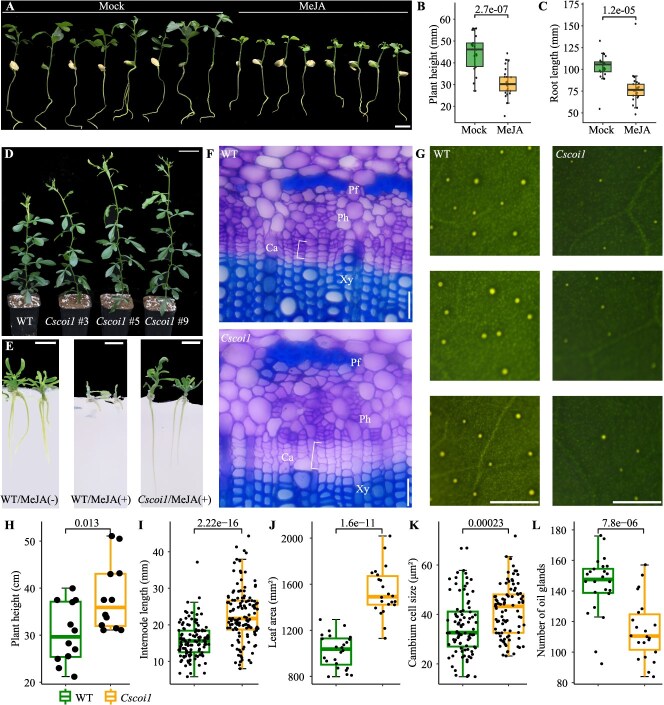
Jasmonate responses in Carrizo Citrange (*Citrus sinensis* 'Washington' sweet orange × *Poncirus trifoliata*) and morphological phenotypes of wild-type (WT) and *Cscoi1* mutants. (A) WT plants after 14 days of mock or methyl jasmonate (MeJA) treatment. Plant heights (B) and root lengths (C) of these plants are shown (n = 25, MeJA; n = 22, mock). (D) Three-month-old WT plants and *Cscoi1* mutants. (E) WT plants and *Cscoi1* mutants after 7 days of mock or MeJA treatment. (F) Representative transverse sections of stems of WT plants and *Cscoi1* mutants. Ph, phloem; Pf, phloem fiber; Ca, Cambium; Xy, xylem. (G) Oil glands in the leaves of WT plants and *Cscoi1* mutants. (H-L) Box plots showing the plant heights (n = 12), internode lengths (n = 120), leaf area (n = 24, WT; n = 22, *Cscoi1*), number of oil glands per 1 cm^2^ of leaves (n = 24, WT; n = 22, *Cscoi1*), and cambium cell size (n = 90) of WT plants and *Cscoi1* mutants. P values from Student’s t test are indicated in B, C, and H-L. Bars, (A) 2 cm; (D) 5 cm; (E) 1 cm; (F) 25 μm; (G) 0.1 cm.

To explore the functions of JA in regulating plant growth in citrus, we treated 1-month-old *in vitro* seedlings of Carrizo Citrange (*Citrus sinensis* 'Washington' sweet orange × *Poncirus trifoliata*) with methyl jasmonate (MeJA). MeJA treatment significantly repressed the growth of both shoots and roots of Citrange seedlings (Fig. 1A–C), suggesting that JA negatively regulates plant growth in citrus. To understand how JA signaling regulates plant growth of citrus, we identified the putative citrus JA receptor through a BLAST search with the protein sequence of *Arabidopsis* COI1, the known JA receptor that plays central role in JA signaling [31]. A single COI1 ortholog was identified in the sweet orange (*Citrus sinensis*) genome. Phylogenetic analysis showed that CsCOI1 shared high sequence similarity with the COI1 proteins in other dicots and was more distant from the COI1 proteins in monocots (Supplementary Fig. S1A).

To examine the functions of the identified CsCOI1, we generated CRISPR mutants of *CsCOI1* in the Carrizo Citrange background. We obtained six homozygous knockout mutants containing frameshift mutations (Fig. 1D and Supplementary Fig. S1B). Whole-genome sequencing of the *Cscoi1*#3 line confirmed no off-target mutations or gene mutations caused by T-DNA insertion. Unless otherwise specified, the *Cscoi1*#3 line was used in the following experiments and referred to as *Cscoi1*. To test the function of CsCOI1 in JA signal perception, WT plants and *Cscoi1* mutants were propagated and treated with MeJA after 2 days of root regeneration. WT plants at this stage showed stronger responses to MeJA treatment. Not only was the root growth inhibited but also the leaves were etiolated or even detached, suggesting a possible role of JA in regulating leaf senescence in citrus (Fig. 1E and Supplementary Fig. S1C). However, none of these phenotypes were present in *Cscoi1* mutants (Fig. 1E and Supplementary Fig. S1C), indicating the crucial role of CsCOI1 in JA signal perception. Consistent with the insensitivity to MeJA, the shoot growth of *Cscoi1* mutants was enhanced (Fig. 1D). Plant heights of 3-month-old *Cscoi1* mutants were significantly higher than those of WT (Fig. 1D and H). Meanwhile, *Cscoi1* mutants had significantly longer internodes (Fig. 1D and I) and larger leaves (Fig. 1J and Supplementary Fig. S1D). Furthermore, cambium cell size in the stems of *Cscoi1* mutants was significantly increased (Fig. 1F and K). These data suggest that JA signaling negatively regulates plant growth in citrus and is dependent on CsCOI1.

### CsCOI1 is a JA receptor

**Figure 2:**
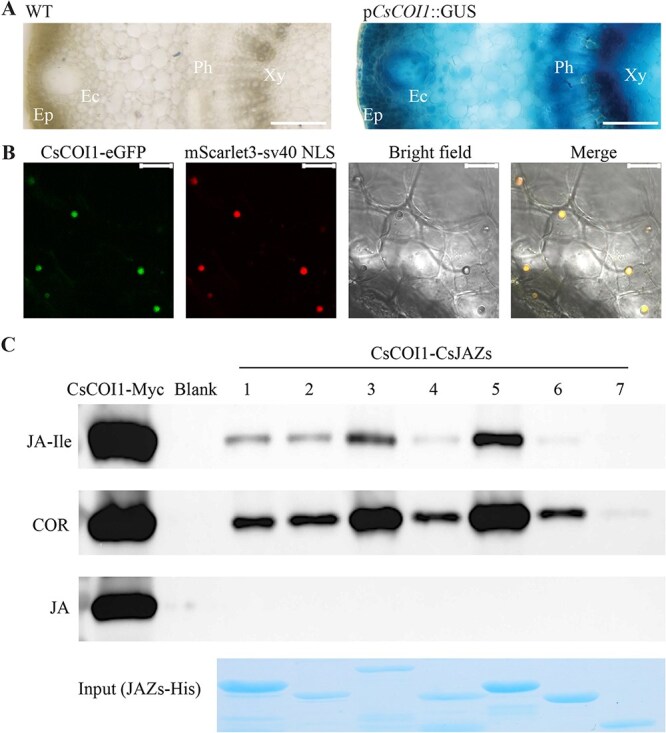
Expression patterns of *Citrus sinensis* CORONATINE INSENSITIVE 1 (CsCOI1) and its interactions with *Citrus sinensis* JASMONATE ZIM DOMAIN (CsJAZ) proteins in the presence of different jasmonates and coronatine (COR). (A) GUS staining signals driven by the *CsCOI1* promoter in the stem of Citrange (*Citrus sinensis* 'Washington' sweet orange × *Poncirus trifoliata*). Ep, epidermis; Ec, epithelial cells of oil glands; Ph, phloem; Xy, xylem. (B) Subcellular localization of CsCOI1. Fluorescence signals were observed in stem sections of stable transgenic Citrange. (C) Pull-down assays showed the interactions between CsCOI1 and CsJAZs. Total protein extracts from CsCOI1-Myc overexpressing lines were subjected to pull-down assays with CsJAZ-His proteins in the presence of jasmonate (JA), jasmonoyl-l-isoleucine (JA-Ile), and COR, respectively. Western blot analysis was performed to detect CsCOI1-Myc protein using an anti-Myc antibody. The Coomassie Brilliant Blue staining was conducted to monitor the input of CsJAZ proteins. Bars, (A) 100 μm; (B) 15 μm.

To understand how CsCOI1 regulates JA signaling in citrus, we analyzed the expression of *CsCOI1* in the transcriptome database [32]. *CsCOI1* was broadly expressed in all analyzed tissues (leaf, root, ovule, peel, pulp, seed; Supplementary Fig. S2A). To further investigate the expression pattern of *CsCOI1*, we generated promoter-reporter lines (p*CsCOI1*::*GUS*) in Citrange. Consistent with the RNA-seq data, GUS staining indicated strong *CsCOI1* expression in all tested tissues (Fig. 2A and Supplementary Fig. S2B). In the stems, the GUS signals were stronger in the xylem and epithelial cells in the oil glands (Fig. 2A). Similarly, in leaves, stronger GUS signals were observed in the vascular bundles (Supplementary Fig. S2B). To examine the subcellular localization of CsCOI1, we generated stable transgenic Citrange lines expressing CsCOI1-eGFP under the control of the *35S* promoter. In the stems of these transgenic plants, CsCOI1-eGFP localized to the nucleus, as evidenced by co-localization with a nuclear marker (mscarlert3-sv40) (Fig. 2B). In addition, the same nuclear localization of CsCOI1 was also observed in *Nicotiana benthamiana* (Supplementary Fig. S2C). This localization is consistent with COI1 proteins in other plants [18, 21] and aligns with the molecular role of CsCOI1 in transcriptional regulation.

In herbaceous plants, COI1 forms a co-receptor complex with JAZ proteins to mediate JA signaling in the presence of bioactive JA [8, 18, 21]. JAZ proteins belong to a larger TIFY gene family. To identify citrus JAZs, we conducted a BLAST analysis using *Arabidopsis* TIFY sequences and identified 14 TIFY-like proteins in the sweet orange genome. The phylogenetic tree showed that 7 of these proteins were putative citrus JAZ proteins (Supplementary Fig. S3A), named CsJAZ1–7. Since the conserved JA-associated (Jas) domain in the JAZ proteins is necessary and sufficient for constructing the COI1-JAZs complex [33], we analyzed the Jas domain of CsJAZs and compared them with AtJAZs. A conserved basic residue (R205 in AtJAZ1) in the Jas domain has been shown to be essential for interaction with AtCOI1 [31, 34]. We found this residue was conserved in CsJAZ1/3/4/5/6, while it was changed in CsJAZ2 and CsJAZ7. CsJAZ2 had a proline substitution at the position corresponding to R205 in AtJAZ1, while CsJAZ7 had a methionine (P179 in CsJAZ2 and M99 in CsJAZ7 correspond to R205 in AtJAZ1) (Supplementary Fig. S3B).

To investigate if CsCOI1 can perceive JA signals in citrus, we performed pull-down assays to assess interactions between CsCOI1 and identified CsJAZs. Since JA-Ile is the effective JA signal in all analyzed flower plants so far [35], we suspected the function of JA-Ile is conserved in citrus. Moreover, coronatine (COR) is a known structural analogue of JA-Ile with equal or much stronger ability to promote the interaction between COI1 and JAZs [36]. Therefore, we examined if JA-Ile acts as an effective ligand for the construction of CsCOI1-CsJAZs co-receptor complex using COR as a control. The Citrange plant-expressed Myc-tagged CsCOI1 and recombinant CsJAZs fused with the His tag were incubated with JA-Ile, COR, and JA, respectively. The pull-down results showed that CsJAZ1–6 directly interacted with CsCOI1 in the presence of JA-Ile, but not when JA was used, while CsJAZ7 only showed weak interaction with CsCOI1 when incubated with COR (Fig. 2C). CsJAZ7 shares a high similarity with AtJAZ8, including a methionine residue at the position corresponding to R205 in AtJAZ1 (Supplementary Fig. S3A and B). AtJAZ8 did not interact with AtCOI1 even when a higher concentration (25 μM) of JA-Ile was used [37], which likely explains the low affinity between CsJAZ7 and CsCOI1. Interestingly, CsJAZ2, which contains a proline at this position, still interacted with CsCOI1, indicating that sequence variation at this site may influence interaction differently depending on the substituted amino acid or may be adapted by different COI1 proteins. These results collectively support that CsCOI1 functions as a JA receptor in citrus and JA-Ile is the bioactive form of JA signal promoting the construction of CsCOI1-CsJAZs co-receptor complex.

### JA signaling promotes resistance to insects in citrus

**Figure 3:**
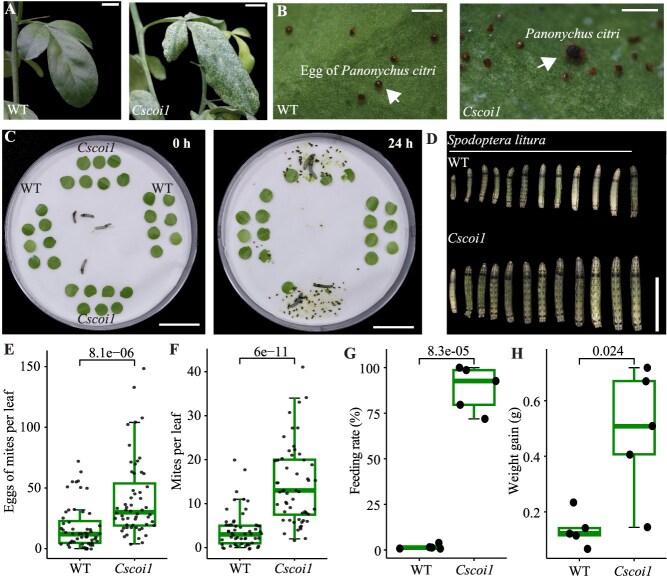
Responses of wild-type (WT) plants and *Cscoi1* mutants to insect feeding. (A) Leaves of WT plant and *Cscoi1* mutant after 45-day challenge of spider mites (*Panonychus citri*). (B) The citrus spider mites and their eggs on leaves of WT plants and *Cscoi1* mutants. The number of eggs (E) and mature mites (F) per leaf were counted after 45 days of challenge, 56 leaves of WT plants and *Cscoi1* mutants were measured, respectively. (C) A picture of leaf discs of WT plants and *Cscoi1* mutants eaten by tobacco cutworm (*Spodoptera litura*) larvae. (D) Representative *S. litura* larvae feeding on WT and *Cscoi1* leaves. (G) Feeding rate of *S. litura* larvae after 24 hours placing (n = 5). (H) Total weight gain of 3 larvae after 7 days feeding on WT and *Cscoi1* leaves (n = 5). P values in Student’s t test are indicated in (E-H). Bars: (A) 1 cm; (B) 0.5 mm; (C) 2 cm; (D) 2 cm.

One key defense mechanism for plants against insects is the elicitation of JA responses [2]. We therefore investigated the role of JA signaling in resistance to insects in citrus. We compared the responses of *Cscoi1* mutants and WT plants under attack by piercing-sucking and chewing insects. The citrus red spider mites (*Panonychus citri*) are key piercing-sucking pests that spread in all citrus-producing areas, causing fruit scarring and help virus invasion [38]. After a 45-day spider mites challenge, the leaves of *Cscoi1* mutants were bleached due to severe mites feeding and their propagation, while fewer lesions were found on WT leaves (Fig. 3A and B). Egg deposition and adult populations of mites on *Cscoi1* mutants were also 4.25-fold and 2.90-fold higher, respectively, than on WT plants (Fig. 3E and F).

Similarly, the tobacco cutworm (*Spodoptera litura*), a chewing pest, showed a preference for feeding on *Cscoi1* mutant leaves over WT leaves. Within 24 hours, 88.57% of leaf discs from *Cscoi1* mutants were consumed, compared to only 1.63% of WT discs (Fig. 3C and G). Correspondingly, larvae feeding on *Cscoi1* mutant leaves gained significantly more weight than those feeding on WT leaves (Fig. 3D and H). Together, these results demonstrate that CsCOI1 promotes resistance to insects and highlight its role in mediating the trade-off between growth and defense against insects in citrus.

### CsCOI1 mediates growth-defense trade-off through transcriptional reprogramming

**Figure 4:**
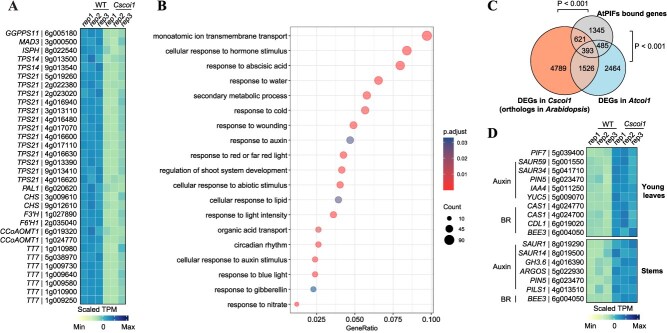
*CsCOI1* mediates transcriptional reprogramming in citrus. (A) Heatmap showing the expression of differentially expressed genes (DEGs) in terpene and flavonoid biosynthesis pathways in young leaves. (B) Enriched gene ontology terms of upregulated genes (*Cscoi1* versus WT) in *Cscoi1* mutants. All genes upregulated in either stems or leaves of *Cscoi1* mutant were analyzed. (C) Venn diagram showing overlapping genes/orthologs of targets of *PHYTOCHROME-INTERACTING FACTORS* (*PIFs*) and DEGs (*coi1* versus WT) in *Cscoi1* and *Atcoi1* mutants. P-values from hypergeometric test are indicated. (D) Heatmap showing the expression of upregulated DEGs in auxin- and brassinosteroid-related pathways in *Cscoi1* mutants. Normalizations for heatmaps were performed using R function scale.

Our results indicate that CsCOI1 mediates a trade-off between growth and biotic stress responses in citrus. To investigate the underlying transcriptional basis, we performed RNA-seq analyses of young leaves and developing stems of WT and *Cscoi1* mutants. We performed differential expression analysis (*Cscoi1* versus WT) and identified 3473 differentially expressed genes (DEGs). We found CsJAZ1–6 were significantly downregulated in *Cscoi1* mutants (Supplementary Table S3), supporting a negative feedback loop in the JA response in citrus and consistent with the abolished JA signaling in *Cscoi1* mutants. Accordingly, many defense-related genes were downregulated in *Cscoi1* mutants. In citrus, essential oils containing terpenes and flavonoids are synthesized and stored in oil glands. These metabolites are known to deter insect herbivores [39, 40] and contribute to the resistance to other biotic stresses [41]. We found that many genes involved in isoprenoid and sesquiterpene biosynthesis were strongly repressed in *Cscoi1* mutants (Fig. 4A). Among 24 expressed (TPM ≥ 1) genes in the terpene synthase (TPS) gene family, 17 were significantly downregulated (Fig. 4A and Supplementary Table S3). Furthermore, genes associated with flavonoid biosynthesis were also downregulated in *Cscoi1* mutants (Fig. 4A). Additionally, we found *CsMYC5* (Cs_ont_5g050360), a gene involved in the later development of oil glands [42], was significantly downregulated in *Cscoi1* mutants (Supplementary Table S3). Correspondingly, the number of oil glands was significantly reduced in *Cscoi1* mutants compared to WT, whereas it was increased in *CsCOI1* overexpressing lines (Fig. 1G and L, Supplementary Fig. S4D and G). In addition, resistance to tobacco cutworm was enhanced in *CsCOI1*-overexpressing lines (Supplementary Fig. S4E, F, H, and  I). Consistent with these phenotypes and the RNA-seq data, some insect-deterring terpenes [43], such as α-pinene and β-myrcene, accumulated at significantly higher levels in *CsCOI1*-overexpressing lines but were reduced in *Cscoi1* mutants compared to WT (Supplementary Fig. S5).

Conversely, many genes related to growth promotion were upregulated in *Cscoi1* mutants. The upregulated DEGs in *Cscoi1* mutants were enriched in gene ontology (GO) terms associated with light responses and auxin responses, similar to *Atcoi1* mutants (Fig. 4B, Supplementary Table S3). Supporting this result, known PIF targets identified in *Arabidopsis* [44] significantly overlapped with upregulated DEGs in both *Cscoi1* and *Atcoi1* mutants (Fig. 4C). Additionally, *PIF7* and genes involved in the response to or biosynthesis of auxin and brassinosteroid were upregulated in *Cscoi1* mutants (Fig. 4D), consistent with the function of PIFs in regulating genes related to these pathways to promote growth [45, 46]. Together, these results suggest that *CsCOI1* mediates transcriptional reprogramming to regulate the growth-defense trade-off in citrus.

### Transcriptional antagonism between JA and SA signaling in citrus

**Figure 5:**
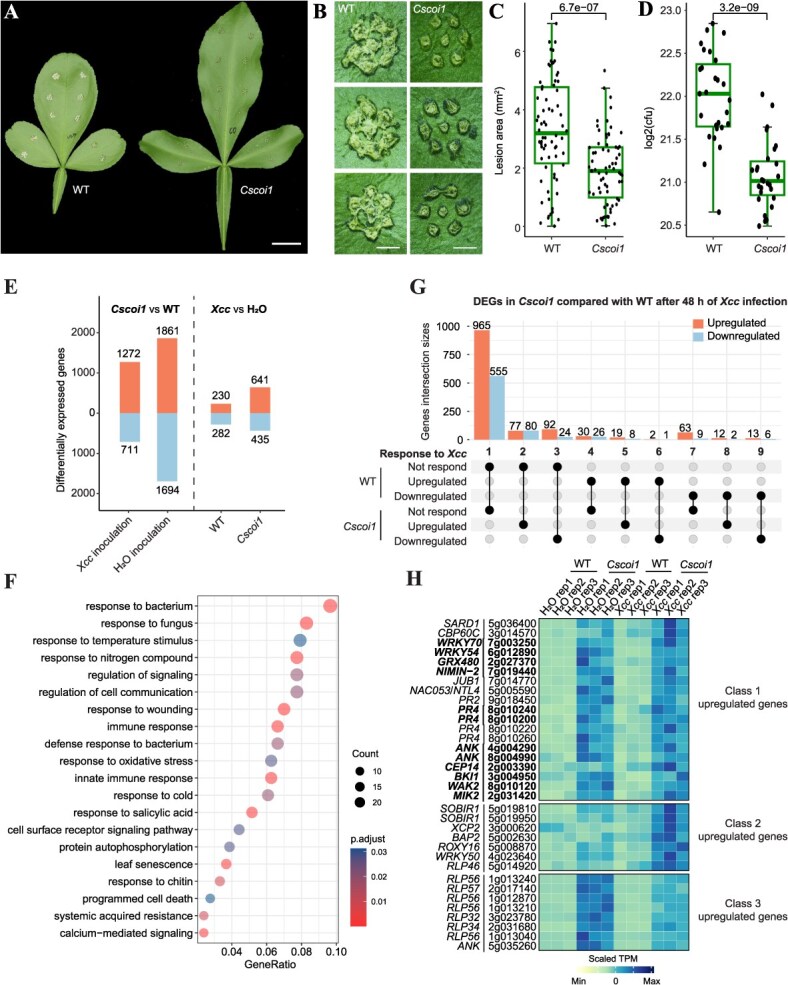
CsCOI1 promotes susceptibility to citrus canker bacterium (*Xanthomonas citri*, *Xcc*). (A) Leaves of wild-type (WT) and *Cscoi1* after 7 days of *Xcc* infection. (B) Magnified symptoms of *Xcc* infection on leaves of WT and *Cscoi1*. (C) Lesion sizes on leaves of WT and *Cscoi1* (n = 70). (D) *Xcc* growth on WT and *Cscoi1* leaves (n = 27). (E) Number of differentially expressed genes (DEGs) in each comparison. (F) Gene ontology terms enriched in upregulated DEGs in *Cscoi1* mutant compared to WT under *Xcc* infection. (G) Upset plot showing the responsiveness to *Xcc* of DEGs identified by comparing gene expression levels between the *Xcc*-inoculated and water-inoculated conditions (*Cscoi1*-*Xcc* versus *Cscoi1*-mock, WT-*Xcc* versus WT-mock). (H) Heatmap showing the expression patterns of representative genes in Class 1, 2, and 3. Normalization was performed using R function scale. Genes marked in bold were *Xcc*-responsive and upregulated in *Xcc* resistant primitive citrus, *Atalantia buxifolia*. P-values in Student’s t test are indicated in (C) and (D). Bars, (A) 1 cm; (B) 1 mm.

In addition to its role in insect defense, JA signaling is involved in pathogen responses and is often associated with the plant hormone SA [47]. To explore the possible crosstalk between JA and SA in citrus pathogen defense, we compared the susceptibility of WT plants and *Cscoi1* mutants to *Xanthomonas citri* subsp. *citri* (*Xcc*), a major bacterium causing citrus canker disease. Our analysis showed that canker-like symptoms developed at significantly fewer inoculation sites on *Cscoi1* mutant leaves (33.3%, 70/212) compared to WT leaves (65.7%, 151/230). Lesion size and bacterial growth were also significantly reduced in *Cscoi1* mutants (Fig. 5A–D). These results suggest that CsCOI1-dependent JA signaling promotes the susceptibility to *Xcc* in citrus.

To gain insight into how CsCOI1 promotes susceptibility to *Xcc*, we analyzed the transcriptomes of *Xcc* infected WT and *Cscoi1* leaves and performed differential expression analysis (*Cscoi1*-*Xcc* versus WT-*Xcc*). A total of 1272 genes were upregulated, and 711 genes were downregulated in *Cscoi1* mutants compared to WT (Fig. 5E). Consistent with the reduced susceptibility of *Cscoi1* mutants, the upregulated DEGs were enriched in GO categories such as response to pathogens and SA, immune system process, and regulation of signaling (Fig. 5F). Correspondingly, core genes in SA signaling were strongly upregulated in *Cscoi1* mutants, including *SARD1*, *CBP60g*, *WRKY70,* and *WRKY54*, with gene expression levels showing 5.04-, 3.45-, 11.20-, and 14.08-fold increases, respectively (Fig. 5H and Supplementary Table S4). Consistent with these gene expression responses, the SA content in the *Cscoi1* mutants was also significantly higher than that in WT plants after 48 hours of *Xcc* inoculation (Supplementary Fig. S6A). In addition, other immune-related genes were also significantly upregulated in *Cscoi1* mutants (Fig. 5H). Furthermore, we noticed that these SA-related genes were not upregulated in *Cscoi1* mutants compared to WT in untreated leaves (Supplementary Table S4), suggesting that the differential expression of these genes was stimulated by inoculation and repressed by CsCOI1.

Next, we investigated gene responses to *Xcc* in citrus by differential expression analysis (*Cscoi1*-*Xcc* versus *Cscoi1*-mock, WT-*Xcc* versus WT-mock). Almost all citrus cultivars, including Carrizo Citrange (the WT used in this study), are highly susceptible to *Xcc* [48]. Thus, to include more responsive genes, we also analyzed our previously published transcriptome data conducted with a *Xcc* resistant primitive citrus, *Atalantia buxifolia* [30]. Genes differentially expressed in response to *Xcc* inoculation compared to water inoculation were determined as responsive. Among responsive genes, only a few (97/1461, 7%) were shared between WT and *Cscoi1* (Supplementary Fig. S6B and Supplementary Table S4). According to responsiveness to *Xcc* infection, we classified the DEGs identified from comparison between *Xcc* inoculated WT and *Cscoi1* leaves (Fig. 5G and H). Class 1 upregulated genes were highly expressed in *Cscoi1* but were not responsive to *Xcc* (Fig. 5G and H). However, we found that 120 of these genes were responsive to *Xcc* and upregulated in *Atalantia buxifolia*, including *WRKY70*/*54*, *PRs*, and other immune-related genes (Supplementary Fig. S6C and Supplementary Table S4). Collectively, these data suggest that CsCOI1 affects the susceptibility to *Xcc* by repressing SA-related genes in broad stimulative processes.

## Discussion

JAs play important roles in regulating the trade-off between growth and defense and have been correlated with many developmental processes that are important for the fruit tree and woods production [11, 49]. Here, we identified the citrus JA receptor, CsCOI1*,* and showed its function in regulating plant growth-defense trade-off (Figs 1–3). *Cscoi1* mutant showed MeJA insensitivity, and our pull-down assays showed that CsCOI1 interacted with CsJAZ1–6 in the presence of JA-Ile, demonstrating that CsCOI1 is the JA receptor in citrus (Figs 1 and 2). These results reveal core components for JA perception in citrus.

A long-standing question is how plants integrate multiple environmental and developmental events while converting them into different outputs through the same JA signaling pathway [16, 50]. Citrus, like *Arabidopsis* and tomato (*Solanum lycopersicum*) [19, 20], contains a single *COI1* gene in their genome (Supplementary Fig. S1A). Therefore, the multiple biological responses to JAs in these plants may be orchestrated by the expanded JAZ gene family through spatial expression difference and specific protein–protein interactions that regulate genes in particular biological processes [51]. Interestingly, we found that CsJAZ2, which contains a non-canonical Jas domain, also interacts with CsCOI1 (Fig. 2C). Notably, the Jas domain of CsJAZ2 is well conserved in citrus genera but diverged in citrus-related genera (Supplementary Fig. S3C). Since the Jas domain is also essential for the interaction of JAZ proteins with many TFs^33^, the altered Jas domain of CsJAZ2 may enable the formation of novel interactions, thereby regulating specific traits in citrus. Moreover, the relatively small JAZ gene family in the citrus genome also provides an opportunity to understand how JA fine-tune multiple phenotypes in woody plants. Furthermore, although only a single *CsCOI1* gene is present, its promoter activity is spatially different, which is higher in the xylem and the epithelial cells of the oil glands (Fig. 2A). Correspondingly, JA signaling is known to positively regulate the development of xylem [52] and oil glands (Fig. 1G, Supplementary Fig. S4D). Although these two organs have distinct functions, programmed cell death (PCD) is necessary for both of their development [53, 54]. Since PCD is also correlated with high JA response [55, 56], we propose that plants may also leverage the same JA-related cell processes for different purposes by transcriptionally fine-tuning the expression of *COI1*.

*Cscoi1* mutants showed enhanced growth, but with the penalty being significantly more susceptible to both piercing-sucking and chewing insects (Figs 1 and 3). Citrus deploys oil glands in the epidermis to defend against biotic stress [41, 42], which are functionally similar to trichomes formed in many other plants [19, 40, 57]. We showed that *CsCOI1*-mediated JA signaling not only promotes the development of oil glands but also the expression of genes related to biosynthesis of terpenes and flavonoids (Figs 1G and 4A), main metabolites of essential oils stored in the oil glands, which are important for the insect resistance [39, 40]. In addition, citrus essential oils are important materials for food and fragrance products. Previous study has shown that the overexpression of *CsDRNL* resulted in increased density of oil glands, while the development of plants was severely disrupted [42]. The overexpression of *CsCOI1*, however, did not cause drastic development abnormalities (Supplementary Fig. S4A). Thus, the production of citrus essential oils may be enhanced through regulation of CsCOI1 and JA signaling. Furthermore, JA signaling also regulates other processes critical for citrus breeding and fruit production, such as phase change [58], flower development [59], flower defense [60], and fruit development [61]. Future studies addressing these aspects will be facilitated by the availability of *CsCOI1*-overexpressing lines and *Cscoi1* mutants.

It was suspected the antagonism between JA and SA might not be present in woody plants. In poplar (*Populus nigra*), study showed that genetically modified poplar with elicited endogenous SA activated the accumulation of JA, while plant growth was not affected even in source limited condition [62]. However, here we demonstrated that the growth of citrus is greatly hindered by exogenous MeJA treatment, while it is enhanced when JAs perception was blocked (Fig. 1), highlighting a critical regulatory role of JAs in plant growth in citrus. In *Cscoi1* mutants, core genes related to SA biosynthesis and signaling, such as *SARD1*, *CBP60g*, and *WRKY70*/*54*, were highly upregulated under defense conditions, demonstrating that the antagonism between JA and SA is present in citrus at the gene expression level (Fig. 5H). Therefore, JA responses and their crosstalk with SA may be distinct in different woody plants and studies focusing on the role of SA in citrus growth control will help clarify its function in regulating the growth-defense trade-off in woody plants.

The antagonistic regulatory relationship between JA and SA confers flexibility to plants in responding to various pathogens, while also posing a risk of manipulation by some pathogens [63]. The reduced susceptibility to *Xcc* exhibited by *Cscoi1* mutants is reminiscent of the response of *Atcoi1* mutants to the bacterial pathogen *Pseudomonas syringae* pv*. tomato* (*Pst*) *DC3000* [64]. *Pst* DC3000 is capable of releasing COR, a structural analog of JA-Ile, to hijack the plant JA signaling pathway for repressing the plant SA-related immune pathway [65]. Several studies have reported that some bacteria in the *Xanthomonas* genus can also produce COR or its analogs [66, 67]. Thus, it would be interesting to test whether *Xcc* can produce COR. Furthermore, it is known that mechanical damages caused by hurricane or insect feeding exacerbate the infection of citrus canker disease [68]. Given that both wounding and insect attacks stimulate JA signaling [49], the antagonism between JA and SA in citrus may facilitate *Xcc* invasion under these conditions. Therefore, it is possible to control the outbreak of canker disease under these conditions by downregulating the JA signaling pathway.

## Conclusion

The work presented here provides insights into how JA signaling regulates the growth-defense trade-off in citrus and enriches our understanding of the JA perception mechanism in woody plants. The *Cscoi1* mutants and *CsCOI1*-overexpressing lines also provide materials for future studies focusing on other JA-related processes in citrus, such as regeneration, reproduction, and fruit development. Our data will facilitate further studies focused on how JA signaling is fine-tuned for plant fitness in citrus. Our results also provide a strategy for breeding citrus with dwarf tree architecture and high resistance, that is to enhance the JA response while knocking out the known *Xcc* susceptibility gene [69].

## Materials and methods

### Plant materials and growth condition

The citrus rootstock cultivar Carrizo Citrange (*Citrus sinensis* 'Washington' sweet orange × *Poncirus trifoliata*) was used as the wild-type (WT) in this study. Transgenic plants were transferred to soil after one month of *in vitro* growth. Plants were grown in an environmentally controlled room (28°C, 16 h light/8 h dark). Plant height and internode length were measured after three months of growth. Tree height was measured from the shoot tip to the bottom of the stem. Lengths of the first ten internodes (counted from the top) were measured. Photos of mature leaves were taken to measure leaf size using ImageJ software. The number of oil glands in a similar region of leaves was counted.

### Phylogenetic analysis

To identify the putative COI1 and JAZ proteins in the sweet orange (*Citrus sinensis*) genome, protein sequences of *Arabidopsis* COI1 and TIFYs were obtained from TAIR (https://www.arabidopsis.org/) and used for BLASTp searches. Maximum likelihood method and default parameters were used to construct phylogenetic trees with IQ-TREE [70]. The tree files were used for visualization by iTOL (https://itol.embl.de/). Protein sequences used for the phylogenetic analyses are listed in Supplementary Table S1.

### Plasmid constructions and transformation

The *CsCOI1* cDNA, the 3256 bp promoter of *CsCOI1* and coding DNA sequences (CDS) of *CsJAZs* were cloned from sweet orange (*Citrus sinensis*). *35S*::*CsCOI1*–*4* × *Myc* and p*CsCOI1*::*GUSPlus* expression cassettes were constructed with golden gate and infusion clone method with a BsaI-mutated pCAMBIA 1300 plasmid. Gene editing plasmids construction and plant transformation were conducted as previously reported [71]. Two guide RNAs were designed to target the *CsCOI1* exon, however only gRNA 2 effectively induced mutations. The CDS of *CsJAZs* were constructed into pET-32a plasmid using EcoRI/SalI sites with infusion cloning methods for production of the JAZs-His protein. Primers used in this study are listed in Supplementary Table S2.

### MeJA treatment

One-month-old *in vitro* seedlings of WT plants were transferred to MS medium containing MeJA (Sigma, 100 μM) or its solvent (DMSO). Plant height and root length were measured 14 days after treatments. WT plants and *Cscoi1* mutants were transferred to MS medium containing MeJA or its solvent (DMSO) 2 days after root regeneration. Total root length was measured on Day 0 and Day 7 after treatment.

### Microscopy and histological staining

Stable transgenic Citrange lines harboring *35S*::*CsCOI1*-*eGFP* and *35S*::*mScarlet3*-*sv40* expression cassettes were generated. Stem sections of these lines were obtained with a vibratome (Leica, VT1000s, Germany) and observed for subcellular localization using a Leica TCS SP8 inverted microscope. The excitation wavelengths for enhanced green fluorescent protein (eGFP) and mScarlet3 [72] were 488 and 552 nm, respectively, and emissions were detected at 505 to 550 nm (eGFP) and 590 to 640 nm (mScarlet3).

GUS staining was performed as previously reported with minor change [73]. Briefly, 1-month-old p*CsCOI1*::*GUSPlus* Citrange plants were used for GUS staining. Tissues were fixed with ice-cold acetone for 2 hours. Acetone was removed, and tissues were rinsed three times with phosphate-buffered saline (PBS). Tissues were then incubated with GUS staining solution (0.2 M NaH_2_PO_4_, 0.2 M Na_2_HPO_4_, 2 mM K_3_[Fe(CN)_6_], 2 mM K_4_[Fe(CN)_6_], and 1 mM X-Gluc) at 37°C for 30 minutes. The GUS signals of roots and leaves were directly observed after immersing in a 75% ethanol for 2 hours to destain. Stem sections of 50 μm thickness were made by vibratome (Leica, VT1000s, Germany), the sections were photographed with an Olympus BX63 microscope (Olympus, Tokyo, Japan).

Three-month-old WT plants and *Cscoi1* mutants were prepared for cambium cell size observation, with six independent replicates. The sixth internodes (counted from the top to the bottom) of each plant were collected. Stem sections of these lines were obtained using a vibratome (Leica, VT1000s, Germany) and stained with 0.05% toluidine blue O solution. Photographs were taken with an Olympus BX63 microscope (Olympus, Tokyo, Japan). Five sections were observed for each plant.

### Pull-down assays

At least 0.1 g leaves of the *35 s*::*CsCOI1*–*4* × *Myc* Citrange overexpression lines were collected and ground in liquid nitrogen for the extraction of total proteins. Total proteins of plant were extracted by incubating the leaves in 1 mL lysis buffer (25 mM Tris–HCl, pH 7.5; 150 mM NaCl; 1 mM EDTA; 10% (v/v) glycerol; 1% (v/v) NP-40; 30 mM Imidazole; 1 mM DTT; 1 mM PMSF; 20 μM MG-132) for 1 hour. The Ni Sepharose beads (25 μL) were washed with lysis buffer. A total of 5 μg CsJAZs-His proteins were incubated with Ni Sepharose beads in 500 μL lysis buffer for 1 hour. For co-immunoprecipitation, the total protein extracted from leaves was incubated with the CsJAZs-His bound Ni Sepharose beads in 500 μL lysis buffer for an additional 1 hour, along with 10 μM JA (GLPbio), 10 μM JA-Ile (GLPbio), 7.5 μM coronatine (Sigma), respectively. The Ni Sepharose beads were washed with lysis buffer, then boiled at 100°C for 10 minutes. Western blot analysis was performed using an anti-Myc tag antibody (Abclonal, China).

### Assay of pathogenicity of citrus canker bacteria

The *Xanthomonas citri* subsp*. citri* (Xcc) strain 3213 was grown on nutrient agar medium at 28°C. Mature leaves of WT plants and *Cscoi1* mutants were inoculated with *Xcc* (10^6^ to 10^8^ CFU/mL) with inoculating needles (0.5 mm in diameter). Six pricks were made at each inoculation site. Bacterial suspension was dropped onto each inoculation site. Sterilized water was used as the control. After 7 days of inoculation, the disease lesion area was measured using ImageJ and leaf discs of three inoculated sites was mixed as one sample for total DNA extraction. Quantitative polymerase chain reaction (qPCR) was conducted with 100 ng of total DNA per sample as the template. *pthA* of *Xcc* was used to quantify the bacterial growth as previously reported [30].

### Insect bioassay

WT plants and *Cscoi1* mutants of the same age were used for red spider mite (*Panonychus citri*) feeding. WT plants and *Cscoi1* mutants were placed separately. The same number of mites were transferred to WT plants and *Cscoi1* mutants. The numbers of mites and eggs were counted after 45 days of challenge. Young leaves from WT plants and *Cscoi1* mutants were collected for feeding the larvae of tobacco cutworm (*Spodoptera litura*). Three larvae were placed in each replicate.

### Whole-genome sequencing

Genomic DNA was extracted from mature leaves of WT and *CsCOI1* genome-edited lines and sequenced on the Illumina HiSeq sequencing platform (Personal Biotechnology, Shanghai, China). Adapters and low-quality reads were removed using Trim Galore. The clean reads were mapped using BWA-MEM. Possible off-target mutations and T-DNA insertions were checked manually.

### Transcriptome analysis

The stems (18 days old), young leaves (7 days old), and untreated and inoculated mature leaves (48 hours after inoculation with *Xcc* or sterilized water) of *Cscoi1* and WT plants, with three biologically independent replicates, were collected for RNA sequencing on the Illumina HiSeq sequencing platform (Personal Biotechnology, Shanghai, China). Published raw RNA-seq data of *Arabidopsis coi1* mutants [63, 74, 75] and *Atalantia buxifolia* [30] were downloaded from the European Nucleotide Archive (ENA). The Col-PEK [76] was used as the reference genome for *Arabidopsis* RNA-seq analysis. The third version genome (SWO.v3.0) of sweet orange and second version genome (HKC.v2.0) of *Atalantia buxifolia* were used for citrange and *Atalantia buxifolia* (http://citrus.hzau.edu.cn/) RNA-seq data analysis, respectively. Adapters and low-quality (Q < 20) reads in raw data were removed using Trim Galore (https://github.com/FelixKrueger/TrimGalore). The clean reads were used for quantification of gene level expression combining Salmon [77] and Tximport [78]. Genes that were not expressed in any condition (average of TPM of three replicates <1) were filtered out. Differentially expressed genes (DEGs) were identified through the R package DESeq2 [79]. The same criterion (absolute value of log_2_ fold change > 0.5 and adjusted p-value < 0.01) was used for identification of DEGs in all comparisons. The expression levels of *CsCOI1* in RNA-seq data were obtained from the CPBD database (http://citrus.hzau.edu.cn/).

Orthologs of *Arabidopsis* and sweet orange were identified using OrthoMCL online tool (https://orthomcl.org/orthomcl/app/workspace/map-proteins/new). Genes in the same OrthoMCL group were regarded as orthologs. The overlap between *Arabidopsis* and citrange DEGs was determined by orthologs present in the same group.

### Metabolites quantification

The volatile metabolites were extracted from 200 mg leaf powder with solid phase microextraction method (40°C, 20 min) and then detected by a gas chromatography–mass spectrometry (GC–MS) system (Agilent 8890/5977C). The oven temperature program was as follows: an initial temperature of 60°C, increased by 6°C/min to 100°C, then further increased by 4°C/min to 240°C, with a final hold for 2 minutes. A split ratio of 20:1 was applied. All volatile metabolites were identified by the NIST public database with retention indices of n-alkanes C7–C40 mixed standards. Using Qualitative Analysis 10.0 software, peak area of each volatile metabolite was calculated, and the relative content of each volatile metabolite was quantified by the peak area of each volatile metabolite, peak area and concentration of nonanoic acid methyl ester (internal standard). For the quantification of SA, metabolites were extracted overnight at 4°C with 1.0 mL 70% of aqueous methanol from 100 mg freeze-dried leaf powder and detected using liquid chromatography-electrospray ionization-tandem mass spectrometry system as previously reported [80]. The extracts were filtered (0.22 μm pore size) before LC–MS analysis. The relative content of salicylic acid was quantified by the peak area.

## Supplementary Material

Web_Material_uhaf174

## Data Availability

Raw sequencing data have been deposited in NCBI and National Genomics Data Center (NGDC, https://ngdc.cncb.ac.cn/). The BioProject numbers in NCBI are PRJNA1216034 (RNA-seq) and PRJNA1216035 (WGS). The BioProject numbers in NGDC are PRJCA035634 (RNA-seq) and PRJCA035635 (WGS).
