# Supplementary material for: CsCOI1 regulates plant growth and defense in citrus
Source: Hortic Res. 2025 Jul 7;12(10):uhaf174. doi: 10.1093/hr/uhaf174 (PMC12528651; doi:10.1093/hr/uhaf174)
Supplement: Web_Material_uhaf174 [file web_material_uhaf174.zip › supplementary figures CsCOI1 regulates plant growth and defense in citrus--R1.docx]

**CsCOI1 regulates plant growth and defense in citrus**

**Supplementary Figures**


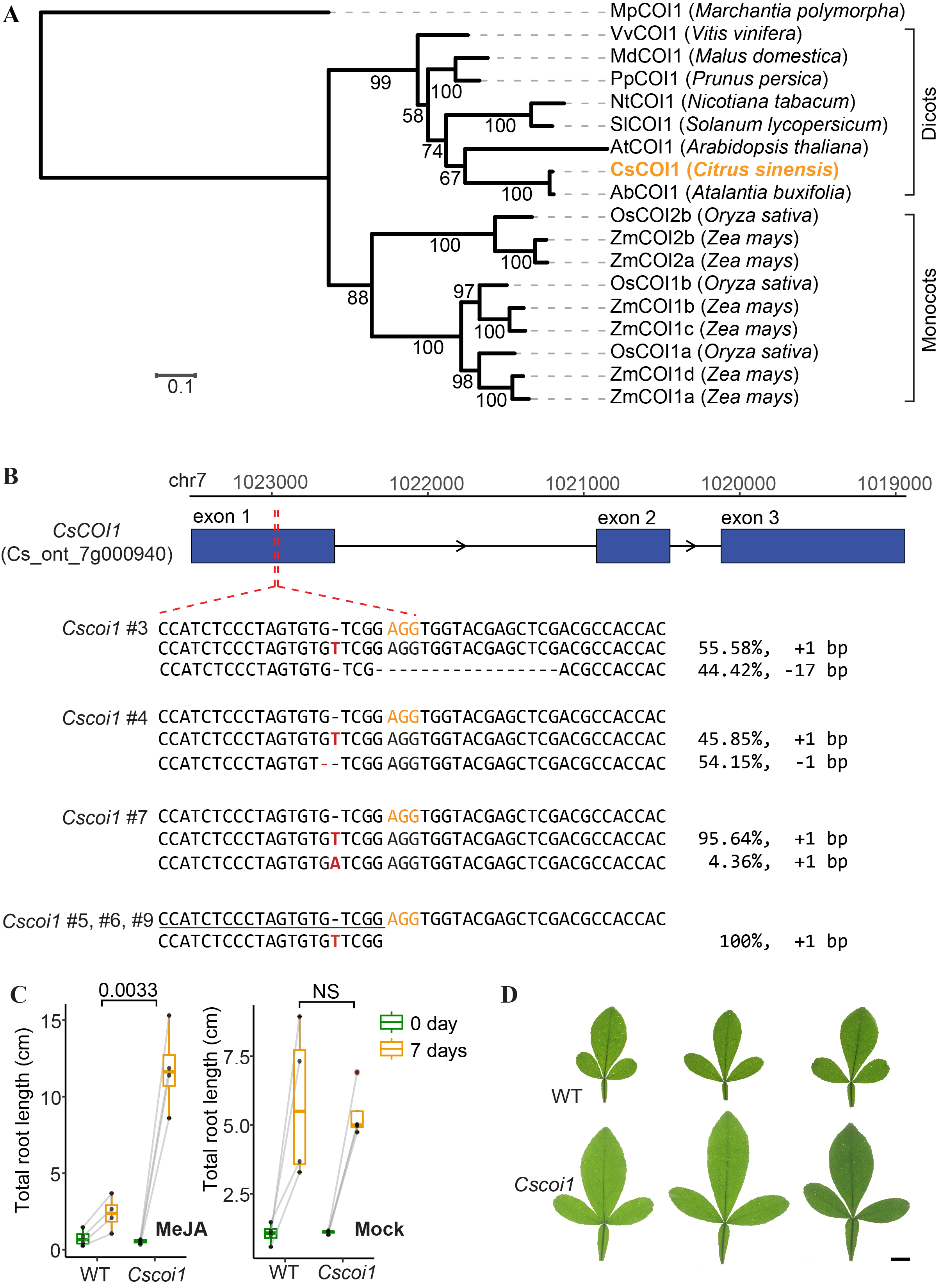


**Figure S1** Phylogenetic analysis and genome editing of *CsCOI1*. (A) Phylogenetic tree of CsCOI1 and COI1 proteins from other plants. The protein sequences are listed in Table S1. (B) Gene structure of *CsCOI1* and CRISPR-Cas9 induced mutation types of each line are shown. The underlined sequence indicates the target of guide RNA. (C) Total root growth of *Cscoi1* mutants and wild-type plants after 7 days of methyl jasmonate treatment. P-value from Student’s t test is indicated. NS, not significant. (D) Leaves of WT and *Cscoi1* mutants. Bar, 1 cm.


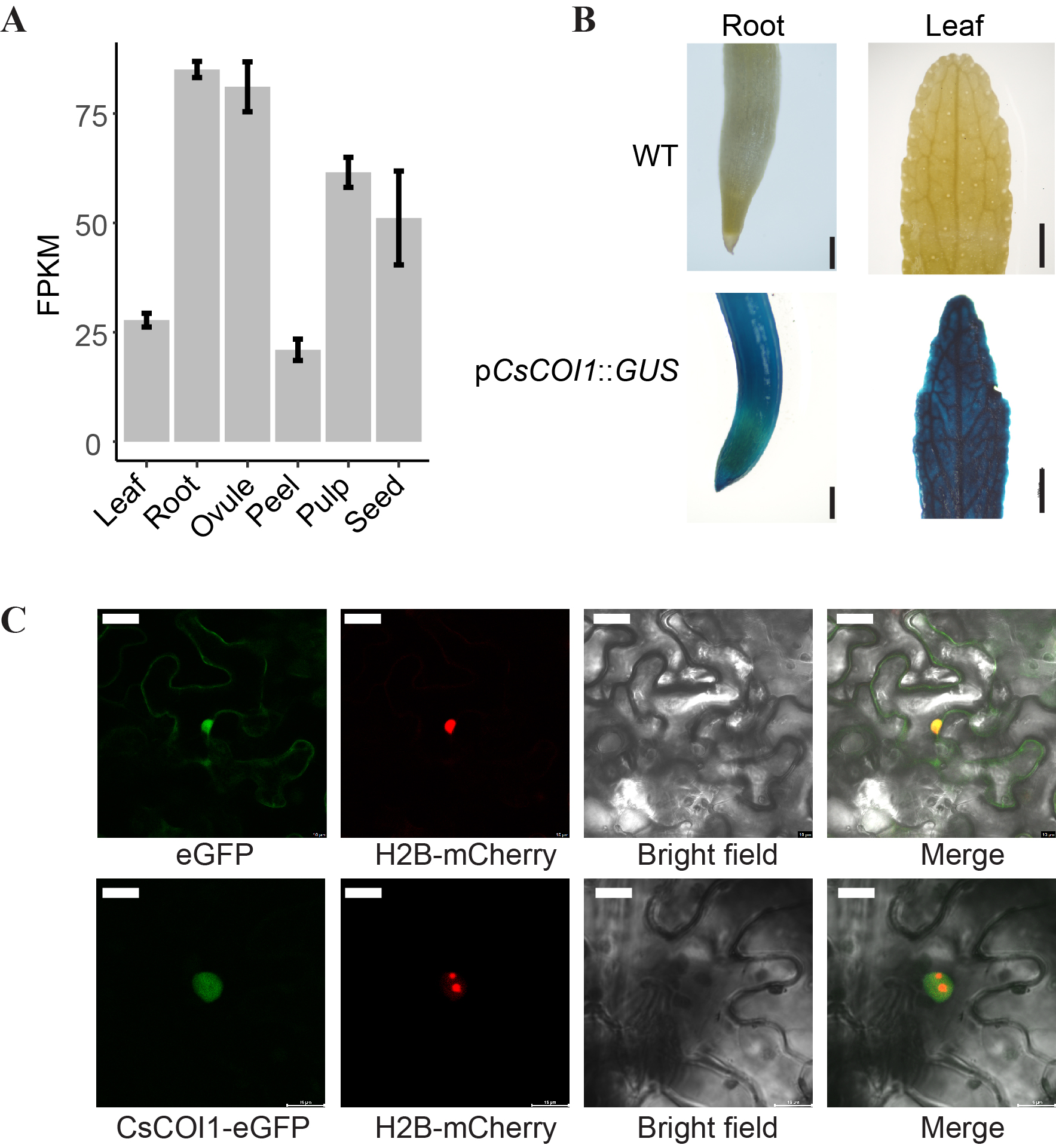


**Figure S2** Expression pattern of CsCOI1. (A) FPKM of CsCOI1 in different tissues of sweet orange (*Citrus* *sinensis*). (B) GUS staining signals driven by the *CsCOI1* promoter in the root and leaf of Citrange (*Citrus sinensis* 'Washington' sweet orange × *Poncirus trifoliata*). Bar, 1 mm. (C) Subcellular localization of CsCOI1. CsCOI1-eGFP was transiently expressed in *Nicotiana benthamiana* leaves. Bar, 15 μm.


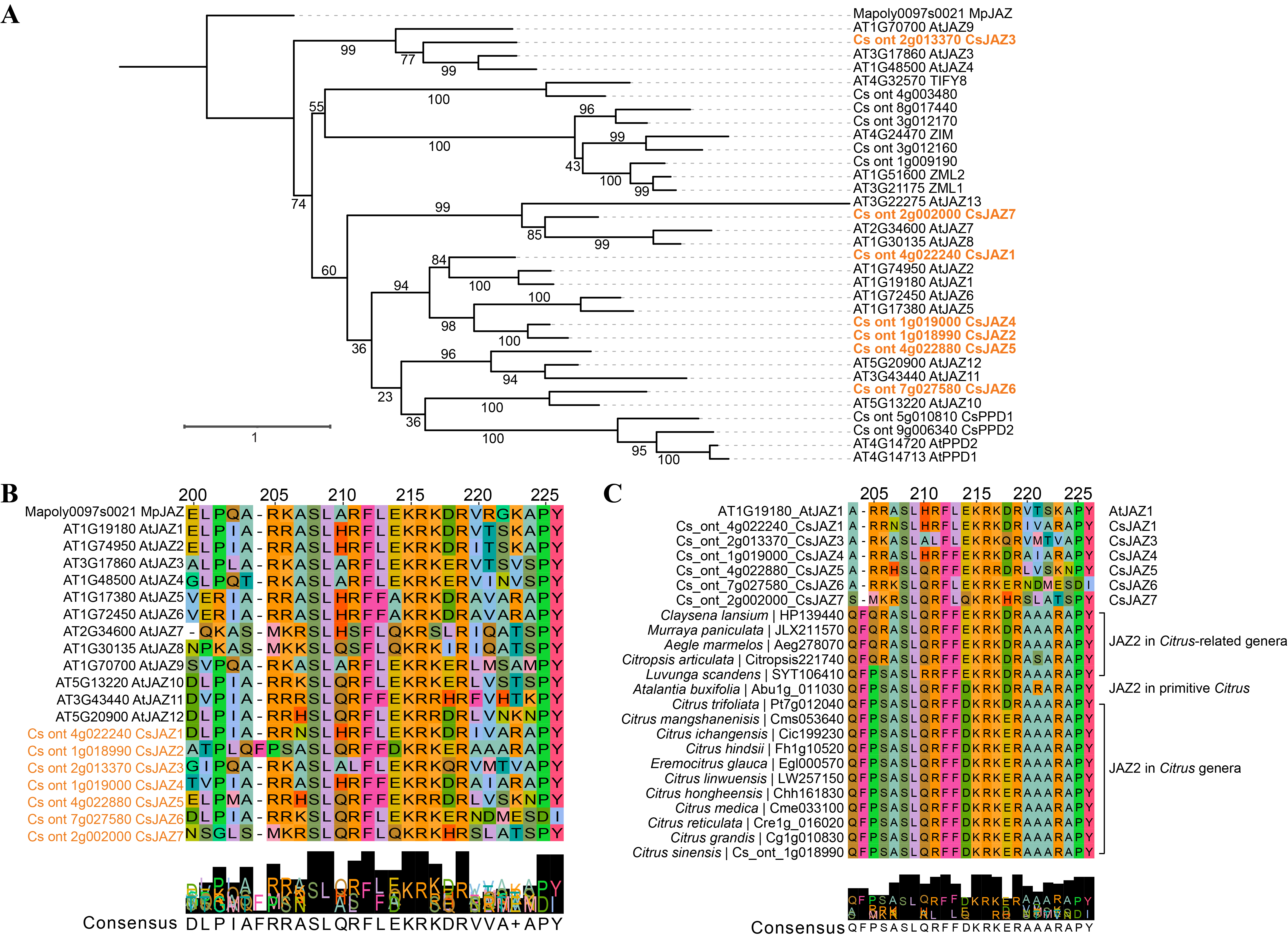


**Figure S3** Phylogenetic analysis of CsJAZs and sequence variation in the Jas domain in CsJAZs. (A) Phylogenetic tree of citrus TIFY gene family. The protein sequences and species names are listed in Table S1. (B) Multiple sequence alignments (MSA) of the Jas motif of *Citrus* *sinensis* and *Arabidopsis* JAZ proteins. The amino acid positions of AtJAZ1 are shown. (C) MSA of the Jas motif of JAZ2 in citrus and its related genera. The amino acid positions of AtJAZ1 are shown.


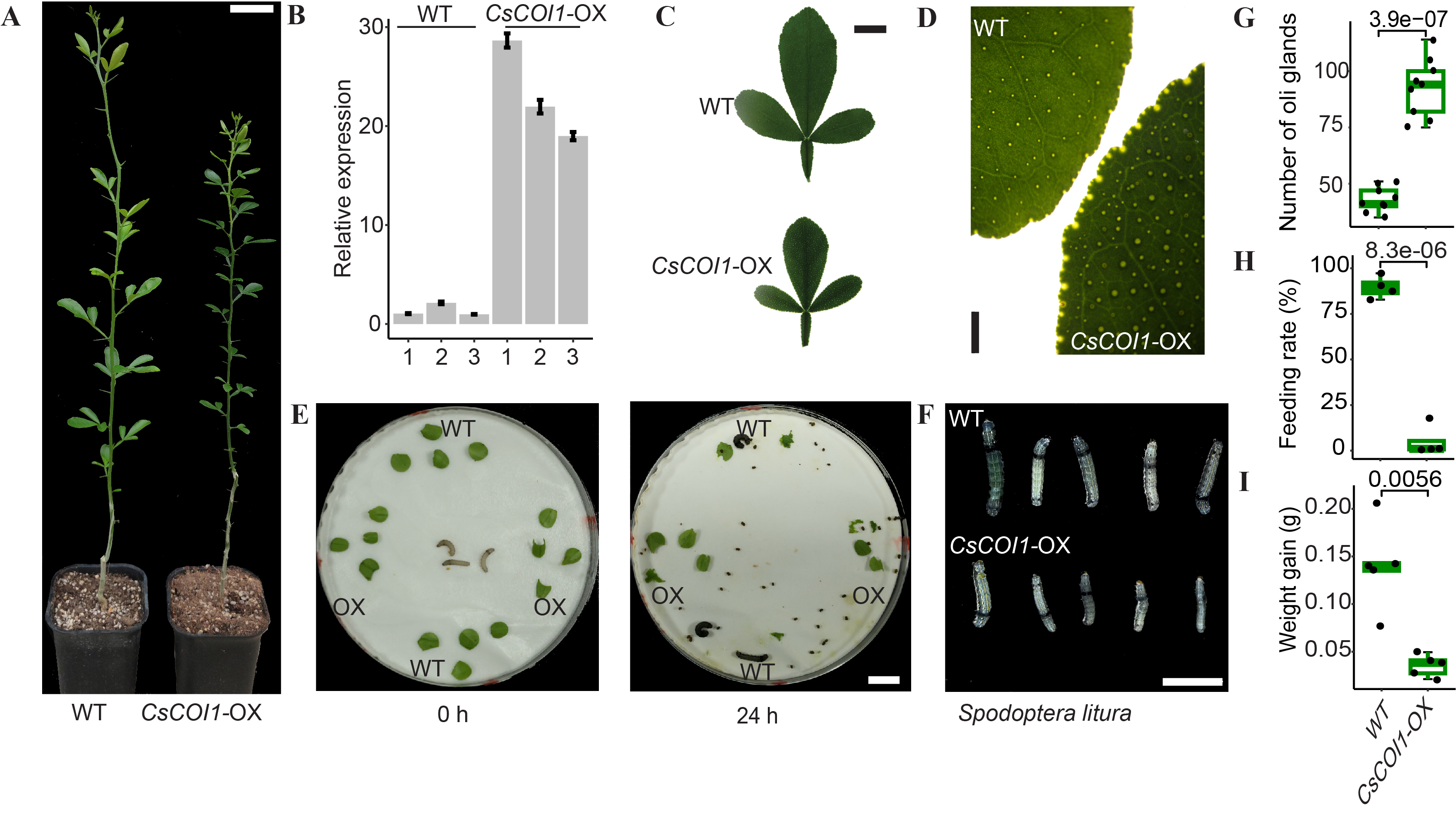


**Figure S4** Overexpression of *CsCOI1* in Carrizo citrange (*Citrus sinensis* 'Washington' sweet orange × *Poncirus trifoliata*). (A) Three-month-old grafted wild-type (WT) plant and *CsCOI1* overexpressing (*CsCOI1*-OX) plant. (B) Relative expression of *CsCOI1* in WT and *CsCOI1*-OX lines. (C) Leaves of WT plants and *CsCOI1*-OX lines. (D) Oil glands on leaves of WT plants and *CsCOI1*-OX lines. (E) A picture of leaf discs of WT plants and *CsCOI1*-OX plants eaten by tobacco cutworm (*Spodoptera litura*) larvae. (F) Representative *S.litura* larvae feeding on WT and *CsCOI1*-OX leaves. (G) Number of oil glands per 0.5 cm^2^ in WT and *CsCOI1*-OX leaves, n = 9. (H) Feeding rate of *S.litura* larvae after 24 h placing, n = 4. (I) Total weight gain of 3 larvae after 7 days feeding on WT and *CsCOI1*-OX leaves, n = 5. P-values in Student’s t test are indicated in (G–I). Bars, (A) 5 cm; (C) 1 cm; (D) 1 mm; (E) 1 cm; (F) 2 cm.


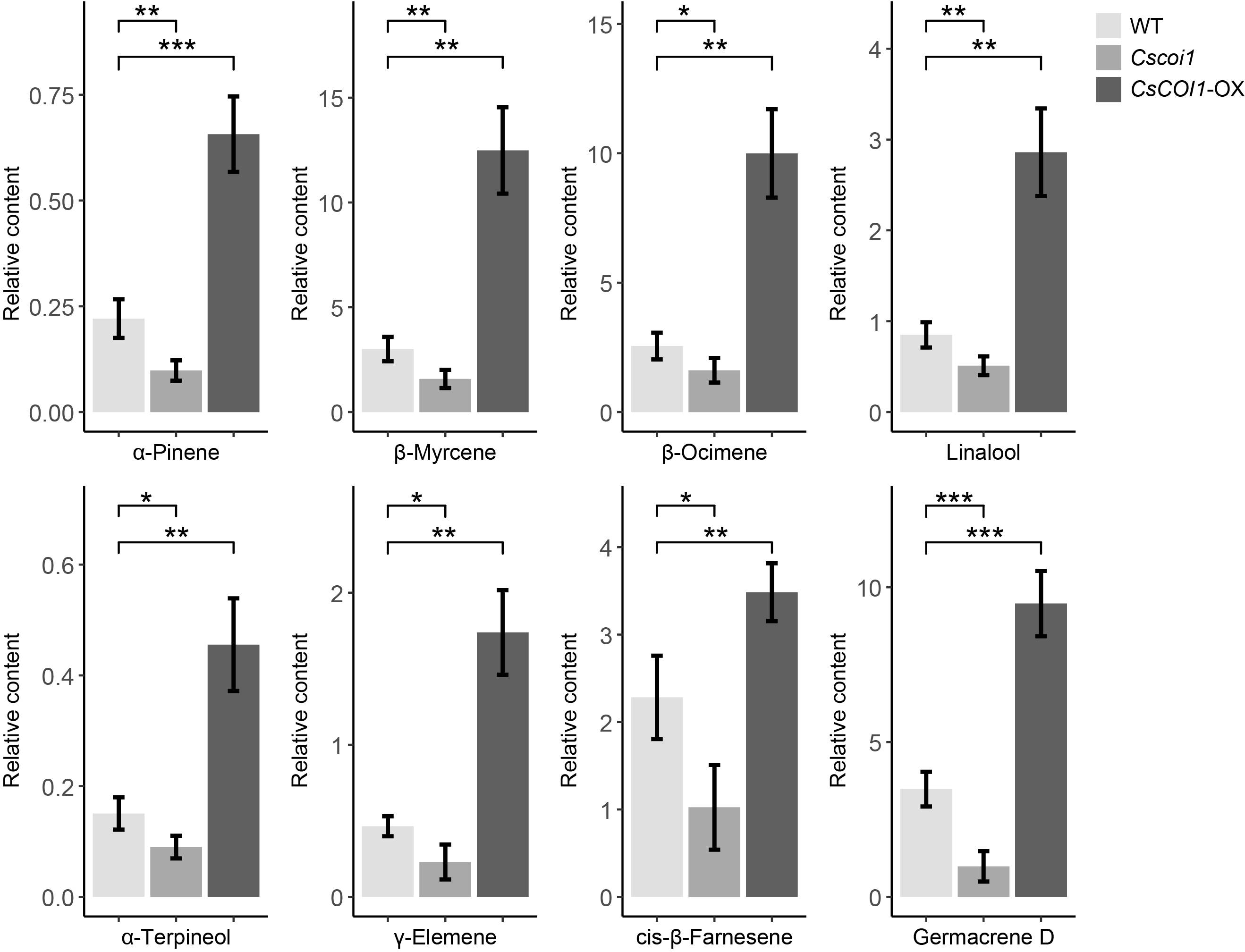


**Figure S5** Relative contents of insect resistance related volatiles in the leaves of wild-type Carrizo citrange (*Citrus sinensis* 'Washington' sweet orange × *Poncirus trifoliata*), *CsCOI1*-OX lines, and *Cscoi1* mutants. Data are means ± standard error (n = 4). P-values (^∗^P < 0.05; ^∗∗^P < 0.01; ^∗∗∗^P < 0.001) in Student’s t test are indicated.


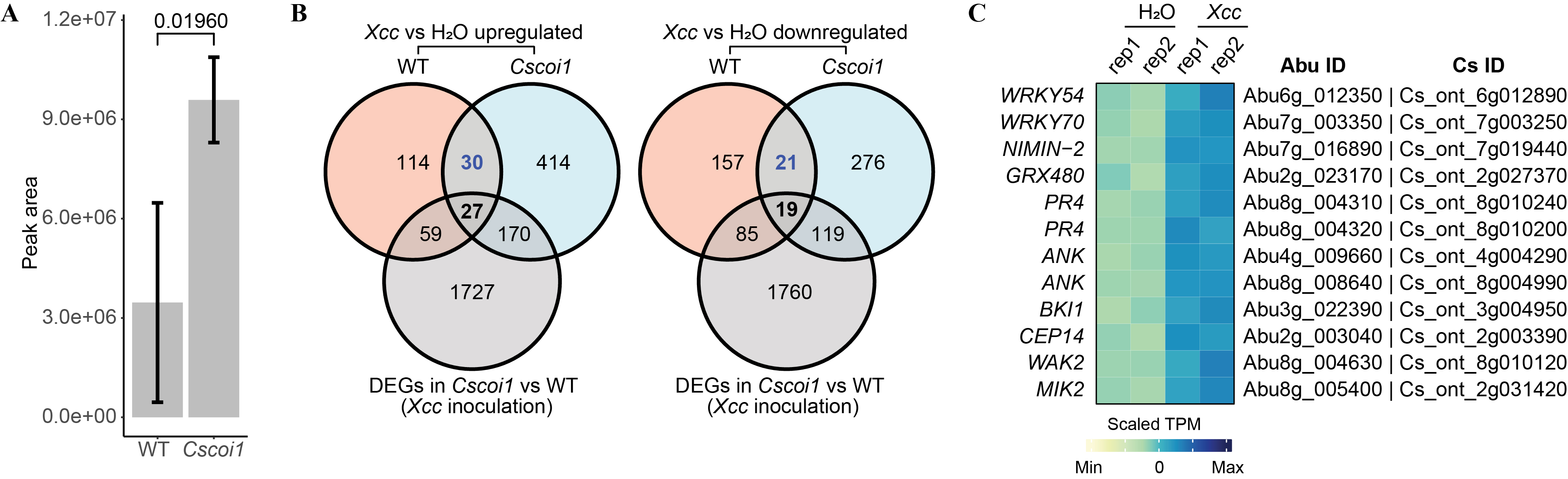


**Figure S6** The responses to *Xcc* in wild-type (WT) Carrizo citrange (*Citrus sinensis* 'Washington' sweet orange × *Poncirus trifoliata*), *Cscoi1* mutants and *Atalantia buxifolia*. (A) The salicylic acid (SA) levels in WT and *Cscoi1* mutants after 48 hours inoculation with *Xcc*. Data are means ± standard error. n =4, WT; n = 3, *Cscoi1* mutant. P-value from Student’s t test is indicated. (B) Venn diagram showing overlapping genes between *Xcc* responsive genes and differentially expressed genes in *Xcc* inoculated WT and *Cscoi1* leaves. (C) Heatmap showing the expression of responsive genes in Class 1 upregulated genes (Fig. 5G) in *Atalantia buxifolia*. Normalization was performed using the R function scale.
